# *Streptomyces colonosanans* sp. nov., A Novel Actinobacterium Isolated from Malaysia Mangrove Soil Exhibiting Antioxidative Activity and Cytotoxic Potential against Human Colon Cancer Cell Lines

**DOI:** 10.3389/fmicb.2017.00877

**Published:** 2017-05-16

**Authors:** Jodi Woan-Fei Law, Hooi-Leng Ser, Acharaporn Duangjai, Surasak Saokaew, Sarah I. Bukhari, Tahir M. Khan, Nurul-Syakima Ab Mutalib, Kok-Gan Chan, Bey-Hing Goh, Learn-Han Lee

**Affiliations:** ^1^Novel Bacteria and Drug Discovery Research Group, School of Pharmacy, Monash University MalaysiaBandar Sunway, Malaysia; ^2^Division of Physiology, School of Medical Sciences, University of PhayaoPhayao, Thailand; ^3^Center of Health Outcomes Research and Therapeutic Safety, School of Pharmaceutical Sciences, University of PhayaoPhayao, Thailand; ^4^Faculty of Pharmaceutical Sciences, Pharmaceutical Outcomes Research Center, Naresuan UniversityPhitsanulok, Thailand; ^5^Department of Pharmaceutics, College of Pharmacy, King Saud UniversityRiyadh, Saudi Arabia; ^6^Department of Pharmacy, Absyn University PeshawarPeshawar, Pakistan; ^7^UKM Medical Molecular Biology Institute, UKM Medical Centre, University Kebangsaan MalaysiaKuala Lumpur, Malaysia; ^8^Division of Genetics and Molecular Biology, Faculty of Science, Institute of Biological Sciences, University of MalayaKuala Lumpur, Malaysia

**Keywords:** *Streptomyces colonosanans*, actinobacteria, mangrove, antioxidant, cancer

## Abstract

*Streptomyces colonosanans* MUSC 93J^T^, a novel strain isolated from mangrove forest soil located at Sarawak, Malaysia. The bacterium was noted to be Gram-positive and to form light yellow aerial and vivid yellow substrate mycelium on ISP 2 agar. The polyphasic approach was used to determine the taxonomy of strain MUSC 93J^T^ and the strain showed a range of phylogenetic and chemotaxonomic properties consistent with those of the members of the genus *Streptomyces*. Phylogenetic and 16S rRNA gene sequence analysis indicated that closely related strains include *Streptomyces malachitofuscus* NBRC 13059^T^ (99.2% sequence similarity), *Streptomyces misionensis* NBRC 13063^T^ (99.1%), and *Streptomyces phaeoluteichromatogenes* NRRL 5799^T^ (99.1%). The DNA–DNA relatedness values between MUSC 93J^T^ and closely related type strains ranged from 14.4 ± 0.1 to 46.2 ± 0.4%. The comparison of BOX-PCR fingerprints indicated MUSC 93J^T^ exhibits a unique DNA profile. The genome of MUSC 93J^T^ consists of 7,015,076 bp. The DNA G + C content was determined to be 69.90 mol%. The extract of strain MUSC 93J^T^ was demonstrated to exhibit potent antioxidant activity via ABTS, metal chelating, and SOD assays. This extract also exhibited anticancer activity against human colon cancer cell lines without significant cytotoxic effect against human normal colon cells. Furthermore, the chemical analysis of the extract further emphasizes the strain is producing chemo-preventive related metabolites. Based on this polyphasic study of MUSC 93J^T^, it is concluded that this strain represents a novel species, for which the name *Streptomyces colonosanans* sp. nov. is proposed. The type strain is MUSC 93J^T^ (= DSM 102042^T^ = MCCC 1K02298^T^).

## Introduction

The discovery of new and useful compounds is constantly in need for the prevention and/or treatment of diseases. Nature has been an interesting source of many useful compounds that have important applications in various fields such as pharmacy, medicine, and biochemistry (Burja et al., 2001; Karikas, 2010). Researchers have been exploring natural sources such as plants and microorganisms for the discovery of novel drugs. Microorganisms have gained increasing attention in drug discovery and many studies revealed that microorganisms from different ecosystems have shown some potentials for human use as many interesting compounds have been derived from them (Burja et al., 2001; Chin et al., 2006).

In the field of microbial drug discovery, *Actinobacteria* strains have been greatly explored due to their ability to produce diverse bioactive secondary metabolites; accounting for 45% of all discovered bioactive microbial metabolites (Sharma and Shah, 2014). Particularly, the dominant genus of this phylum which is *Streptomyces* have a significant contribution to mankind (Azman et al., 2015). The genus *Streptomyces* is proposed by Waksman and Henrici (1943) and it is a group of Gram positive bacteria comprised ~780 species with validly published names (http://www.bacterio.cict.fr/). Members of this genus are producers of more than 75% of the naturally occurring antibiotics (Kinkel et al., 2014; Lee et al., 2014e; Ser et al., 2015a). Other than antibiotics, *Streptomyces* bacteria are prolific producers of various compounds with important biological activities such as antifungal, anticancer, antioxidant, and immunosuppressive activities (Kino et al., 1987; Rashad et al., 2015; Ser et al., 2016a; Law et al., 2017). It is known that exploring new taxa is one of the successful strategies that can lead to the discovery of therapeutic agents (Williams, 2009; Ser et al., 2016c). In previous drug screening programs, it is unfortunate that the screening of novel *Actinobacteria* from terrestrial source have resulted in inefficient rediscovery of known bioactive compounds (Ser et al., 2016c). Therefore, this highlighted the need to discover novel *Actinobacteria* from new or under explored area such as the mangrove environments.

Mangrove environments consists of special woody plant area mainly located in intertidal zones of estuaries, deltas, lagoons, backwaters, creeks, marshes, tropical, and subtropical coastal regions (Mangamuri et al., 2012; Ser et al., 2015a). Mangrove is one of the world's most dynamic environments which occupies millions of hectors across the world coastal areas and it has been a habitat to various flora and fauna of terrestrial, freshwater, and marine species (Mangamuri et al., 2012; Lee et al., 2014e). According to the report by Giri et al. (2011), the largest extent of mangroves is found in Asia; and Malaysia is one of the most mangrove-rich countries in Asia. Additionally, one of the least disturbed mangrove areas in Malaysia is situated at the state of Sarawak, in which most of its mangrove forests are still in pristine condition (Ashton and Macintosh, 2002). Hence, this provides a great opportunity to explore the actinobacterial population present in these mangrove forests.

Owing to the presence of various microbial enzymatic and metabolic activities, the mangrove ecosystem is highly rich in nutrient and organic matter that in turn facilitates the rapid development of species diversity in response to environmental variation (Satheeja and Jebakumar, 2011; Mangamuri et al., 2012). Furthermore, this ecosystem experiences constant fluctuations in salinity and tidal gradient that could trigger metabolic pathway adaptations and possibly lead to the production of pharmaceutically important metabolites. Hence, there are growing interests in the utilization of mangrove microorganism resources and this have subsequently led to the discovery of novel *Streptomyces* (Hong et al., 2009; Lee et al., 2014d,e).

In recent studies, researchers have successfully identified a number of novel *Streptomyces* from mangrove environments in different countries. For examples, *Streptomyces avicenniae* (Xiao et al., 2009), *Streptomyces xiamenensis* (Xu et al., 2009), *Streptomyces sanyensis* (Sui et al., 2011), and *Streptomyces qinglanensis* (Hu et al., 2012) from mangrove environments in China, *Streptomyces sundarbansensis* (Arumugam et al., 2011) from mangrove environments in India, and *Streptomyces pluripotens* (Lee et al., 2014d), *Streptomyces mangrovisoli* (Ser et al., 2015b), *Streptomyces humi* (Zainal et al., 2016), *Streptomyces antioxidans* (Ser et al., 2016c), and *Streptomyces malaysiense* (Ser et al., 2016b) from mangrove environments in Malaysia. In addition, several studies also reported that mangrove *Streptomyces* are capable of producing antioxidant and anticancer agents (Ser et al., 2015a, 2016b,c; Tan et al., 2015). Thus, this prompted the investigation of *Streptomyces* from underexplored mangrove forest in Sarawak.

As a matter of fact, the current global burden of cancer is increasing continuously and this is mainly due to increasing urbanization, followed by the changes in environmental conditions and lifestyle (Karikas, 2010; Ser et al., 2016b; Siegel et al., 2016). Over the years, natural compounds play a relevant role in cancer therapy and prevention (Nobili et al., 2009). Several anticancer agents that have been successfully derived from *Streptomyces* include aclarubicin, bleomycin, doxorubicin, mitomycin C, and pentostatin (Tan et al., 2006, 2015). Besides, the knowledge acquired throughout years of research conducted in cancer biology has emphasized the cancer initiation and progression is mainly associated with oxidative stress- a condition characterized by elevated amounts of free radicals (Reuter et al., 2010; Ser et al., 2016b). Oxidative stress is known to cause modification or damage to important cellular macromolecules including DNA which could dramatically increase the risk of cancer (Reuter et al., 2010; Ser et al., 2015a, 2016b; Tan et al., 2015). Meanwhile, antioxidant is acknowledged to play important role in biological system. It exerts its scavenging ability to neutralize the free radicals and thus preventing deleterious effects of excessive free radicals during occurrence of oxidative stress (Ser et al., 2015a, 2016b). In view of the importance of antioxidant, efforts have been made to search for effective natural antioxidants. A number of antioxidants have been derived from *Streptomyces* such as carazostatin (Kato et al., 1989), antiostatins A_1_ to A_4_ and B_2_ to B_5_ (Mo et al., 1990), carbazoquinocins A to F (Tanaka et al., 1995), and benthocyanins A, B, and C (Shin-ya et al., 1991; Shinya et al., 1993).

This study explores novel *Streptomyces* strains present in soil samples collected from the mangrove forest located at Kuching, Sarawak. A novel strain, MUSC 93J^T^ was discovered and polyphasic approach demonstrated that MUSC 93J^T^ represents a novel species of the *Streptomyces* genus, for which the name *Streptomyces colonosanans* sp. nov. is proposed. With the advancement of next generation sequencing (NGS) technology, the genome of MUSC 93J^T^ was analyzed in this study. The study also aim to investigate the antioxidant and anticancer properties of MUSC 93J^T^. Furthermore, gas chromatography-mass spectrometry (GC-MS) was conducted for chemical analysis of MUSC 93J^T^ extract in order to reveal the active compounds present in the extract. To the best of our knowledge, there is no literature reported so far in regards to the exploration of biological properties of *Streptomyces* isolated from Sarawak mangrove environments. Therefore, the outcome of this study provides a further in depth understanding on bioprospecting potential of *Streptomyces* from under-explored region of Malaysia and at the same time granting the solid foundation to support for a further in depth molecular studies on chemopreventive property possessed by *Streptomyces colonosanans* sp. nov.

## Materials and methods

### Isolation and maintenance of strain

Strain MUSC 93J^T^ was isolated from a soil sample collected at site KTTAS 1 (1°41′48.57′N 110°11′15.30″E), situated in the mangrove forest of Kuching, state of Sarawak, Malaysia, in June 2015. Samples of the upper 20 cm topsoil layer (after the top 2–3 cm of soils removed) were collected using an aseptic metal trowel, placed in sterile Eppendorf tube and stored in −20°C. Air-dried soil samples were ground with a mortar and pestle. Selective pretreatment of soil samples was performed using wet heat in sterilized water (15 min at 50°C; Takahashi et al., 1996). One gram of air-dried soil was mixed with 9 mL sterilized water and then the suspension was spread onto an isolation medium ISP 2 (Shirling and Gottlieb, 1966) supplemented with cycloheximide (50 mg/L) and nalidixic acid (20 mg/L), and incubated at 28°C for 14 days. Pure cultures of strain MUSC 93J^T^ were obtained and maintained on ISP 2 agar slants at 28°C and stocked in glycerol suspensions (20%, v/v) at −20°C.

### Genomic and phylogenetic analyses

**Figure 1:**
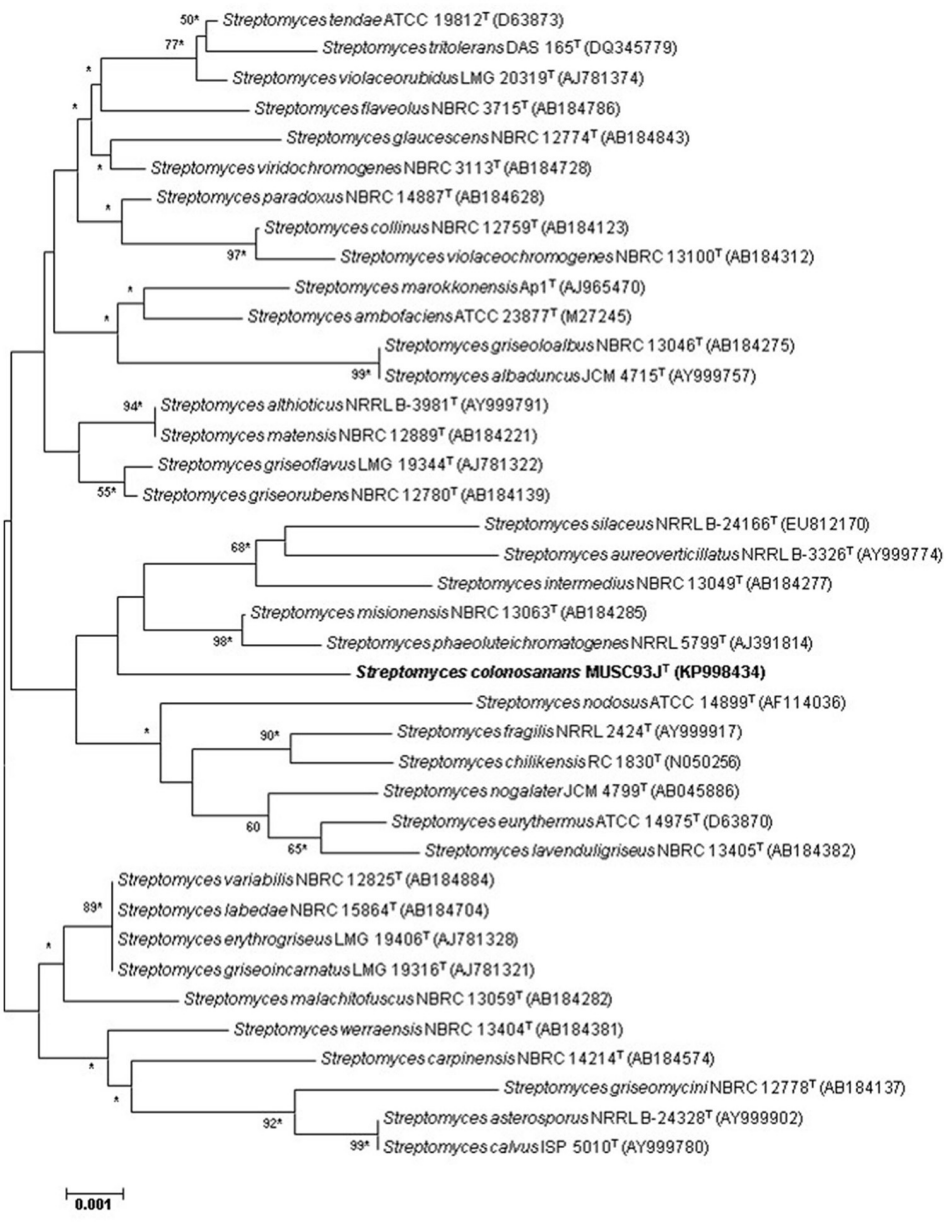
**Neighbor-joining phylogenetic tree based on almost complete 16S rRNA sequences (1,490 nucleotides) showing the relationship between strain MUSC 93J^**T**^ and representatives of some other related taxa**. Numbers at nodes indicate percentages of 1,000 bootstrap re-samplings, only values above 50% are shown. Bar, 0.002 substitutions per site. Asterisks indicate that the corresponding nodes were also recovered using the maximum-likelihood tree-making algorithm.

Genomic DNA extraction for PCR was performed as described by Hong et al. (2009). PCR amplification of the 16S rRNA gene was conducted according to the protocol described by Lee et al. (2014d). The 16S rRNA gene sequence of strain MUSC 93J^T^ was aligned with representative sequences of related type strains in the genus *Streptomyces* retrieved from the GenBank/EMBL/DDBJ databases using CLUSTAL-X software (Thompson et al., 1997). The alignment was first verified manually and adjusted, followed by construction of phylogenetic trees with neighbor-joining (Saitou and Nei, 1987; Figure 1) and maximum-likelihood algorithms (Felsenstein, 1981; Figure S1), utilizing the MEGA version 6.0 (Tamura et al., 2013). For neighbor-joining algorithm, the evolutionary distances were computed using the Kimura's two-parameter model (Kimura, 1980). The calculations of level of sequence similarity were performed by EzTaxon-e server (http://eztaxon-e.ezbiocloud.net/; Kim et al., 2012). Bootstrap based on 1,000 resampling method of Felsenstein (1985) was used to analyze the stability of the resultant tree topologies.

For DNA-DNA hybridization, the extraction of genomic DNA of strain MUSC 93J^T^, *Streptomyces malachitofuscus* JCM 4493^T^, *Streptomyces misionensis* NBRC 13063^T^ and *Streptomyces phaeoluteichromatogenes* DSM 41898^T^ were conducted according to the protocol described by Cashion et al. (1977). DNA-DNA hybridization was performed by the Identification Service of the DSMZ, Braunschweig, Germany based on the procedure as described by De Ley et al. (1970) with slight modifications according to Huss et al. (1983). The G + C content of strain MUSC 93J^T^ was determined by HPLC (Mesbah et al., 1989).

BOX-PCR fingerprint analysis was performed for the characterization of strain MUSC 93J^T^ and the closely related strains with the use of primer BOX-A1R (5′-CTACGGCAAGGCGACGCTGACG-3′; Versalovic et al., 1991; Lee et al., 2014b). The BOX-PCR cycling parameters were performed as described by Lee et al. (2014a) and the PCR products were visualized using 2% agarose gel electrophoresis.

### Chemotaxonomic characteristics

Biomass and freeze-dried cells for chemotaxonomic studies were obtained after growing in TSB at 28°C for 5 days on a rotary shaker. The analyses of peptidoglycan amino acid composition and sugars of strain MUSC 93J^T^ were performed by the Identification Service of the DSMZ using published protocols (Schumann, 2011). Analysis of fatty acids (Sasser, 1990), polar lipids (Kates, 1986), and respiratory quinones were performed by the Identification Service of the DSMZ. Major diagnostic whole cell sugars of strain MUSC 93J^T^ were obtained according to the description by Whiton et al. (1985) and analyzed by TLC on cellulose plates (Staneck and Roberts, 1974).

### Phenotypic characteristics

**Figure 2:**
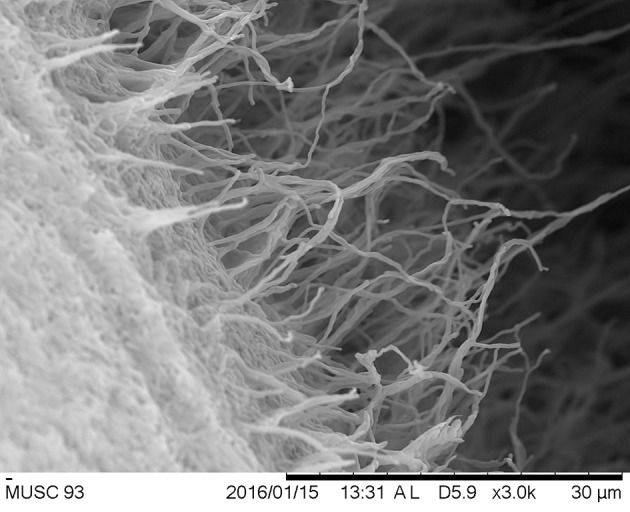
**Scanning electron microscope of ***Streptomyces colonosanans*** MUSC 93J^**T**^**.

The cultural characteristics of strain MUSC 93J^T^ was determined following growth on ISP 2, ISP 3, ISP 4, ISP 5, ISP 6, and ISP 7 agar (Shirling and Gottlieb, 1966), actinomycetes isolation agar (AIA; Atlas, 1993), starch casein agar (SCA; Küster and Williams, 1964), *Streptomyces* agar (SA; Atlas, 1993), and nutrient agar (Macfaddin, 2000) at 28°C for 14 days. The morphology of strain MUSC 93J^T^ was observed after incubation on ISP 2 agar plate at 28°C for 7–14 days (Figure 2), using Light microscopy (80i, Nikon) and scanning electron microscopy (JEOL-JSM 6400). The designations of colony colors were made according to the ISCC-NBS color charts. Gram staining was carried out by standard Gram reaction and confirmed by using KOH lysis (Cerny, 1978). The pH range for growth and NaCl tolerance were evaluated using tryptic soy broth (TSB). The pH range tested was between pH 4.0 and 10.0 at an interval of 1 pH unit. The concentration of NaCl was tested at a range of 0–10% (w/v) at intervals of 2%. The effects of temperatures on growth was examined on ISP 2 agar. The temperature range tested for growth was between 4 and 44°C at intervals of 4°C. The growth responses to pH, NaCl, and temperature were observed for 14 days. The production of melanoid pigments was examined using ISP 7 medium following protocol described by Lee et al. (2014c). Hemolytic activity was examined on blood agar medium containing 5% (w/v) peptone, 3% (w/v) yeast extract, 5% (w/v) NaCl, and 5% (v/v) horse blood (Carrillo et al., 1996). Plates were examined for hemolysis after incubation at 32°C for 7–14 days. Amylolytic, lipase, cellulase, chitinase, catalase, protease, and xylanase activities were determined by growing cells on ISP 2 medium following protocol as described by Lee et al. (2014c). The presence of clear zones around colonies indicates the potential of isolates for surfactant production. The carbon-source utilization and chemical sensitivity assays were determined using Biolog GenIII MicroPlates (Biolog, USA) according to the manufacturer's instructions.

All of the phenotypic assays mentioned were performed concurrently for strain MUSC 93J^T^, *Streptomyces malachitofuscus* JCM 4493^T^, *Streptomyces misionensis* NBRC 13063^T^, and *Streptomyces phaeoluteichromatogenes* DSM 41898^T^.

### Extract preparation of MUSC 93J^T^

Before fermentation process, strain MUSC 93J^T^ was grown in TSB (Biomerge, Malaysia) as seed medium for 14 days. The fermentation medium, Han's Fermentation Media 1 (HFM1) was autoclaved at 121°C for 15 min prior to experiment (Hong et al., 2009; Lee et al., 2012). The fermentation was conducted in 500 mL Erlenmeyer flask containing 200 mL HFM1 (Biomerge, Malaysia) with 200 μL seed media added into it and shaking at 200 rpm for 7–10 days at 28°C. The resulting Han's Fermentation medium was recovered by centrifugation at 12000 g for 15 min. The supernatant was filtered and collected, then subjected to freeze drying process. The freeze-dried sample was extracted with methanol for 72 h. The methanol-containing extract was filtered and collected, then subjected to re-extraction under same condition for twice at 24 h interval. The collected extract was concentrated with extracting solvent evaporated by rotary vacuum evaporator at 40°C. The final concentrate extract of MUSC 93J^T^ was collected and suspended in dimethyl sulfoxide (DMSO) as vehicle reagent prior to bioactivity screening assays.

### Determination of antioxidant activity of MUSC 93J^T^ extract using different assays

#### The 2,2′-azino-bis (3-ethylbenzothiazoline-6-sulfonic acid) (ABTS) assay

The 2,2′-azino-bis (3-ethylbenzothiazoline-6-sulfonic acid) (ABTS) assay was conducted according to the protocol described by Miser-Salihoglu et al. (2013) with some modifications. ABTS radical cation (ABTS·) was produced via reacting ABTS stock solution (7 mM) and potassium persulfate (2.45 mM) for 24 h before the assay. The absorbance was measured at 743 nm and the change in radical amount was indicated by the reduction in absorbance value.

#### Superoxide anion scavenging/superoxide dismutase (SOD)

Superoxide anion scavenging /superoxide dismutase (SOD) activity was determined using SOD assay Kit—WST (Sigma-Aldrich), a commercially available colorimetric microtiter plate method, according to the protocol given by the manufacturer. SOD activity of the extract was determined colorimetrically at 450 nm as the reduction of the Dojindo's highly water-soluble tetrazolium salt, WST-1 (2-(4-iodophenyl)-3-(4-nitrophenyl)-5-(2,4-disulfophenyl)-2H-tetrazolium, monosodium salt) by superoxide anion, ${\text{O}}_{2}^{-}$. Sample extract solution (20 μL) at different concentrations were added to the 96-well-plate, respectively. The reaction solutions were added as described in the protocol and then the plate was incubated at 37°C for 20 min prior to measurement of absorbance at 450 nm using a microplate reader. The percentage of SOD activity (percentage of WST-1 reduction) was calculated as follows (Tan et al., 2015):

$$\begin{array}{l} \text{Percentage of SOD activity }(\%)\\ =\;\frac{\left(\left(\text{Absorbance of blank }1-\text{Absorbance of blank }3\right)-\left(\text{Absorbance of sample}-\text{Absorbance of black }2\right)\right)}{\left(\text{Absorbance of blank }1-\text{Absorbance of blank }3\right)}\;\times \;100\% \end{array}$$

#### Metal chelating

Metal Chelating activity was examined by measuring the formation of Fe^2+^-ferrozine complex as described in previous study conducted by Manivasagan et al. (2013) with slight modification. FeSO_4_ (2 mM) was added into the extract followed by the addition of ferrozine (5 mM) to initiate the reaction prior to measurement of absorbance at 562 nm using spectrophotometer.

### Maintenance and growth condition of human cell lines

The human normal colon CCD-18Co cells were maintained in DMEM media supplemented with 10% fetal bovine serum in a humidified incubator with 5% CO_2_ in air at 37°C (Ser et al., 2016c). All of the human derived cancer cell lines evaluated in this study were maintained in RPMI (Roswell Park Memorial Institute)-1640 (Gibco) supplemented with 10% fetal bovine serum and 1x antibiotic-antimycotic (Gibco) in a humidified incubator with 5% CO_2_ in air at 37°C (Tan et al., 2015).

### Investigation of cytotoxicity activity of MUSC 93J^T^ using 3-(4,5-dimethylthazol-2yl)-2,5-diphenyl tetrazolium-bromide (MTT) assay

For evaluation of cytotoxicity, the human normal colon CCD-18Co cells were included in this study, while the human derived cancer cell lines included were colon cancer cell lines: HCT-116, HT-29, Caco-2, and SW480. The cytotoxic activity of MUSC 93J^T^ extract was examined using MTT assay following the protocol previously described by Williams (1989). The cell viability was determined spectrophotometrically at 570 nm (with 650 nm as reference wavelength) using a microplate reader. The percentage of cell viability was calculated as follows:

$$\begin{array}{l} \text{Percentage of cell viability }(\%)\\ =\;\frac{\text{Absorbance of treated cells}}{\text{Absorbance of untreated cells}}\times 100\% \end{array}$$

### Gas chromatography-mass spectrometry (GC-MS) analysis

GC-MS analysis was performed according to previously developed method with slight modification (Supriady et al., 2015). The analysis was conducted using Agilent Technologies 6980N (GC) equipped with 5979 Mass Selective Detector (MS), with HP-5MS (5% phenyl methyl siloxane) capillary column of dimensions 30.0 m × 250 μm × 0.25 μm and helium as carrier gas at 1 mL/min. The column temperature was programmed initially at 40°C for 10 min, followed by an increase of 3°C/min to 250°C and was kept isothermally for 5 min. The MS was operating at 70 eV. The constituents were identified by comparison of their mass spectral data with those from NIST 05 Spectral Library.

### Genome sequencing and bioinformatics analysis of MUSC 93J^T^

Genomic DNA of MUSC 93J^T^ was extracted using Masterpure™ DNA purification kit (Epicentre, Illumina Inc., Madison, WI, USA) followed by RNase (Qiagen, USA) treatment (Ser et al., 2015c, 2016d) Subsequently, the DNA quality was examined using NanoDrop spectrophotometer (Thermo Scientific, Waltham, MA, USA) and a Qubit version 2.0 fluorometer (Life Technologies, Carlsbad, CA, USA). DNA library construction was performed using Nextera™ DNA Sample Preparation kit (Nextera, USA) and the library quality was validated by Bioanalyzer 2100 high sensitivity DNA kit (Agilent Technologies, Palo Alto, CA). Paired-end sequencing was carried out on MiSeq platform with MiSeq Reagent Kit 2 (2 × 250 bp; Illumina Inc., Madison, WI, USA). The paired-end reads were then trimmed and *de novo* assembled with CLC Genomics Workbench version 7 (CLC bio, Denmark). Gene prediction was carried out using Prodigal version 2.6, whereas rRNA and tRNA were predicted using RNAmmer and tRNAscan SE version 1.21 (Lowe and Eddy, 1997; Lagesen et al., 2007; Hyatt et al., 2010). The assembly was annotated using Rapid Annotation using Subsystem Technology (RAST) and by the NCBI Prokaryotic Genomes Annotation Pipeline (Angiuoli et al., 2008; Aziz et al., 2008). Results of genome sequencing and bioinformatics analysis were presented in the description of *Streptomyces colonosanans* sp. nov.

### Statistical analysis

Experiments involved the investigation of antioxidant and cytotoxic activities were done in quadruplicate. Data analysis was performed with SPSS statistical analysis software and the results were expressed as mean ± standard deviation (*SD*). One-way analysis of variance (ANOVA) followed by appropriate *post-hoc* test (Tukey) was performed to determine the significant differences between groups. A difference was considered statistically significant when *p* ≤ 0.05.

## Results and discussion

### Genomic and phylogenetic analyses of strain MUSC 93J^T^

The nearly complete 16S rRNA gene sequence was determined for strain MUSC 93J^T^ (1490 bp; GenBank/EMBL/DDBJ accession number KP998434) and manual alignment of the sequence was performed with the corresponding partial 16S rRNA gene sequences of the type strains of representative members of the genus *Streptomyces* retrieved from GenBank/EMBL/DDBJ databases. Phylogenetic trees were constructed based on the 16S rRNA gene sequences to determine the phylogenetic position of this strain (Figure 1 and Figure S1). Phylogenetic analysis exhibited that closely related strains include *Streptomyces misionensis* NBRC 13063^T^ (99.1% sequence similarity) and *Streptomyces phaeoluteichromatogenes* NRRL 5799^T^ (99.1% sequence similarity), as they formed a distinct clade (Figure 1). The analysis of 16S rRNA gene sequence for strain MUSC 93J^T^ exhibited highest sequence similarity to strain *Streptomyces malachitofuscus* NBRC 13059^*T*^ (99.2%), *Streptomyces misionensis* NBRC 13063^T^ (99.1%) and *Streptomyces phaeoluteichromatogenes* NRRL 5799^T^ (99.1%); sequences similarities of <99.0% were obtained with the type strains of other species of the genus *Streptomyces*.

The DNA–DNA relatedness values between strain MUSC 93J^T^ and *Streptomyces malachitofuscus* JCM 4493^T^ (14.4 ± 0.1%), *Streptomyces misionensis* NBRC 13063^T^ (46.2 ± 0.4%) and *Streptomyces phaeoluteichromatogenes* DSM 41898^T^ (20.7 ± 1.0%) were significantly below 70%, the threshold value for the delineation of bacterial species (Wayne et al., 1987).

The BOX-PCR results indicated that strain MUSC 93J^T^ exhibited a unique BOX-PCR fingerprint compared with closely related type strains: *Streptomyces malachitofuscus* JCM 4493^T^, *Streptomyces misionensis* NBRC 13063^T^, and *Streptomyces phaeoluteichromatogenes* DSM 41898^T^ (Refer to Figure S2). These results are in line with results of phylogenetic analysis and DNA-DNA hybridizations, which demonstrate that strain MUSC 93J^T^ represents a novel species in the genus *Streptomyces*.

### Chemotaxonomic analyses of strain MUSC 93J^T^

The fatty acids profiles of strain MUSC 93J^T^ and closely related type strains are presented in Table 1. The major cellular fatty acids in MUSC 93J^T^ were identified as anteiso-C_15:0_ (23.1%), C_16:0_ (18.6%), and iso-C_16:0_ (15.1%). The fatty acids profile of MUSC 93J^T^ is consistent with those of closely related phylogenetic neighbors such as *Streptomyces malachitofuscus* JCM 4493^T^, *Streptomyces misionensis* NBRC 13063^T^, and *Streptomyces phaeoluteichromatogenes* DSM 41898^T^, which contain anteiso-C_15:0_ (12.6–40.1%) and iso-C_16:0_ (14.4–18.3%) as major fatty acids (Table 1). However, the fatty acid profile of MUSC 93J^T^ was quantitatively different from those of these type strains; for instance, the anteiso-C_15:0_ (23.1%) was found to be predominant in strain MUSC 93J^T^, but the amount of anteiso-C_15:0_ was much lesser (12.6%) in *Streptomyces malachitofuscus* JCM 4493^T^ (Table 1).

**Table 1:** **Cellular fatty acid composition of strain MUSC 93J^**T**^ and its closely related ***Streptomyces*** species**.

**Fatty acid**	**1**	**2**	**3**	**4**
iso-C_12:0_	0.1	0.1	–	–
C_12:0_	0.1	–	–	–
iso-C_13:0_	0.3	0.4	0.1	0.2
anteiso-C_13:0_	0.3	0.2	0.3	–
iso-C_14:0_	5.4	2.5	1.8	4.8
C_14:0_	1.1	0.4	0.2	0.2
iso-C_15:0_	9.6	17.5	7.2	12.3
anteiso-C_15:0_	23.1	12.6	40.1	35.5
C_15:1_ w6c	0.2	0.1	–	–
C_15:0_	2.3	1.9	0.7	1.6
iso-C_16:1_ H	0.6	2.0	1.6	1.3
iso-C_16:0_	15.1	18.3	14.4	17.7
C_16:1_ Cis 9	–	–	1.3	0.7
C_16:0_	18.6	7.9	4.0	3.4
iso-C_17:1_ w9c	1.3	7.9	–	–
anteiso-C_17:1_ w9c	1.1	2.6	–	–
anteiso-C_17:1_ C	–	–	4.1	2.8
iso-C_17:0_	3.4	8.6	2.4	3.5
anteiso-C_17:0_	7.6	8.8	19.3	13.4
C_17:1_ w8c	0.7	1.2	–	–
C_17:1_ Cis 9	–	–	0.1	0.2
C_17:0_ CYCLO	0.9	0.2	0.4	0.3
C_17:0_	1.6	1.2	0.2	0.2
C_17:0_ 10-Methyl	–	0.3	–	–
iso-C_18:1_ H	–	0.3	–	–
iso-C_18:0_	0.3	0.2	–	–
C_18:1_ w9c	–	0.2	–	–
C_18:1_ w7c	0.2	0.2	–	–
C_18:0_	0.6	0.1	–	–

*Strains: 1, Streptomyces colonosanans MUSC 93J^T^; 2, Streptomyces malachitofuscus JCM 4493^T^; 3, Streptomyces misionensis NBRC 13063^T^; 4, Streptomyces phaeoluteichromatogenes DSM 41898^T^. –, < 0.1% or not detected. All data are obtained concurrently from this study*.

Based on the results of the polar lipids analysis, strain MUSC 93J^T^ showed the presence of aminolipid, diphosphatidylglycerol, lipid, phospholipid, phosphatidylinositol, phosphatidylethanolamine, and phosphoglycolipid. The differences in polar lipid profiles showed that MUSC 93J^T^ is different from related type strains; for example, strain MUSC 93J^T^ contain two aminolipids (Figure S3A), while *Streptomyces malachitofuscus* JCM 4493^T^ only contain one aminolipids (Figure S3B).

The chemotaxonomic analyses also demonstrated that the cell wall of strain MUSC 93J^T^ is of cell-wall type I as it contains LL-diaminopimelic (Lechevalier and Lechevalier, 1970). Many other species of the genus *Streptomyces* were also found to have LL-diaminopimelic (Lee et al., 2005, 2014d; Xu et al., 2009; Hu et al., 2012; Ser et al., 2015b,d). The predominant menaquinones of strain MUSC 93J^T^ were identified as MK-9(H_8_) (42%) and MK-9(H_6_) (35%). This finding is in agreement with the report of the study conducted by Kim et al. (2003), in which the predominant menaquinones of *Streptomyces* are MK-9(H_8_) and MK-9(H_6_). The cell wall peptidoglycan was determined to contain LL-diaminopimelic acid. The whole cell sugars were found to be glucose, mannose, and ribose. The G + C content of strain MUSC 93J^T^ was found to be 69.9 mol%; this is within the range of 67.0–78.0 mol% described for species of the genus *Streptomyces* (Kim et al., 2003).

### Phenotypic analyses of strain MUSC 93J^T^

Strain MUSC 93J^T^ exhibited good growth on ISP 2 agar, ISP 6 agar, and *Streptomyces* agar after 7 days at 28°C, moderate growth on starch casein agar, weak growth on nutrient agar, and no growth on actinomycetes isolation agar, ISP 3, ISP 4, ISP 5, and ISP 7 agar. The 15-day-old culture of strain MUSC 93J^T^ formed light yellow aerial and vivid yellow substrate mycelium on ISP 2 agar (Table 2). These morphological characteristics are consistent with grouping of the strain to the genus *Streptomyces* (Williams, 1989). The NaCl tolerance, temperature ranges, and pH for growth of strain MUSC 93J^T^ occurred at 0–4% (optimum 0–2%), 24–36°C (optimum 0–2%), and pH 6.0–7.0 (optimum pH 6.0), respectively. The cells of MUSC 93J^T^ were positive for catalase and haemolytic activities. Hydrolysis of soluble starch, casein and tributyrin (lipase) were positive; but negative for hydrolysis of chitin, carboxymethylcellulose, and xylan. Based on a range of phenotypic properties, strain MUSC 93J^T^ can be differentiated from closely related members of the genus *Streptomyces* (Table 2). In chemical sensitivity assays, cells are resistant to 1% sodium lactate, D-serine, rifamycin RV, minocycline, lincomycin, niaproof 4, tetrazolium violet, tetrazolium blue, nalidixic acid, potassium tellurite, aztreonam, sodium butyrate, and sodium bromate.

**Table 2:** **Differentiation characteristics of strain MUSC 93J^**T**^ and type strains of phylogenetically closely related species of the genus ***Streptomyces*****.

**Characteristic**	**1**	**2**	**3**	**4**
**MORPHOLOGY (ON ISP 2):**				
Color of aerial mycelium	Light yellow	Pale greenish yellow	Yellowish white	Light yellow
Color of substrate mycelium	Vivid yellow	Vivid greenish yellow	Yellowish gray	Brilliant Greenish yellow
**GROWTH AT**				
28°C	+	(+)	(+)	+
36°C	(+)	+	+	+
pH 6	+	(+)	(+)	(+)
2% NaCl	+	(+)	+	(+)
Catalase	+	+	+	+
Hemolytic	+	−	−	−
**HYDROLYSIS OF**				
Casein (protease)	+	−	−	+
Tributyrin (lipase)	+	−	+	+
Starch (amylolytic)	+	+	+	+
Carboxymethylcellulose (cellulase)	−	+	+	+
Xylan (xylanase)	−	−	+	−
**CARBON SOURCE UTILIZATION**				
D-maltose	−	+	+	+
Sucrose	−	+	+	+
D-turanose	−	+	+	+
D-raffinose	+	−	+	−
α-D-lactose	−	+	+	+
β-methyl-D-glucoside	−	+	−	+
D-salicin	−	+	−	+
N-acetyl-D-glucosamine	−	+	+	+
D-fructose	−	+	+	+
L-rhamnose	−	+	−	+
D-sorbitol	−	+	+	+
myo-inositol	−	+	+	+
Pectin	−	+	+	+
Methyl pyruvate	−	+	+	+
D-lactic acid methyl ester	−	+	+	+
Citric acid	−	+	+	+
α-keto-glutaric acid	−	+	+	+
D-malic acid	−	+	+	+
L-malic acid	−	+	+	+
Bromo-succinic acid	−	+	+	+
Propionic acid	−	+	+	+
Acetic acid	−	+	+	+
Formic acid	−	+	+	+

*Strains: 1, Streptomyces colonosanans MUSC 93J^T^; 2, Streptomyces malachitofuscus JCM 4493^T^; 3, Streptomyces misionensis NBRC 13063^T^; 4, Streptomyces phaeoluteichromatogenes DSM 41898^T^. All data were obtained concurrently in this study. +, Positive; -, negative; (+), weak*.

*All strains are positive for utilization of Dextrin, D-trehalose, D-cellobiose, Gentiobiose, α-D-glucose, D-mannose, D-galactose, glycerol, D-glucose-6-PO4, D-fructose-6-PO4, Gelatin, D-galacturonic acid, L-galactonic acid lactone, D-gluconic acid, D-glucuronic acid, Glucuronamide, p-hydroxy-phenylacetic acid, L-lactic acid, Tween 40, γ-amino-butyric acid, α-hydroxy-butyric acid, β-hydroxy-D,L-butyric acid, α-keto-butyric acid, and acetoacetic acid. All strains are negative for assimilation of 3-methyl glucose*.

According to the outcomes of genomic, phylogenetic, chemotaxonomic and phenotypic analyses, strain MUSC 93J^T^ merits assignment to a novel species in the genus *Streptomyces*, for which the name *Streptomyces colonosanans* sp. nov. is proposed.

### Antioxidant activity of strain MUSC 93J^T^ extract

Oxygen free radicals, also known as reactive oxygen species (ROS) are products of a normal cellular metabolism process in an organism (Valko et al., 2006). Oxidative stress occurs when there is an overproduction free radicals and deficiency of antioxidants, resulting in the accumulation of free radicals (Valko et al., 2006; Reuter et al., 2010). This condition may cause damage to DNA, proteins, and lipids which has been associated with the development of age-related diseases such as cancer, arthritis, and neurodegenerative disorders in living organisms (Valko et al., 2006; Tan et al., 2015). For that reason, antioxidants are required as they may play an important role in preventing the deleterious effects of free radicals and thus they are regard as potential bioactive agents against cancers in human (Kawanishi et al., 2005; Reuter et al., 2010; Ser et al., 2016b). For example, a trial conducted by Blot et al. (1993) in China suggested that combination of antioxidants of beta carotene, vitamin E, and selenium may decrease the risk of gastric cancer. Besides, several studies and meta-analysis of the epidemiological literature have shown that increased intake of lycopene, a potent antioxidant present in tomatoes, is associated with reduced risk of prostate cancer (Gann et al., 1999; Giovannucci et al., 2002; Etminan et al., 2004) and gastric cancer (Yang et al., 2013).

In the early 1980s, the scientific community started to focus on the exploration of microbial antioxidants. Since then, researchers have discovered and characterized a variety of antioxidant compounds from microorganisms in hope to be developed into novel therapeutic agents (Hall, 2001). Due to the pathophysiological complexity of the human diseases, the bioprospecting activities in search for more effective and specific antioxidants from natural resources is still required. In this context, *Streptomyces* bacteria emerges as one of the good sources of natural compounds since they are prolific producers of bioactive secondary metabolites. Furthermore, several studies have reported the detection of compounds with antioxidant property extracted from *Streptomyces* spp., for instance, thiazostatin A and thiazostatin B (Shindo et al., 1989), diphenazithionin (Hosoya et al., 1996), dihydroherbimycin A (Chang and Kim, 2007) as well as 5-(2,4-dimethylbenzyl)pyrrolidin-2-one (Saurav and Kannabiran, 2012).

The present study showed that MUSC 93J^T^ merits assignment to a novel species in the genus *Streptomyces* based on the polyphasic approach analyses. Since the strain MUSC 93J^T^ is a novel *Streptomyces* species, it would be interesting to investigate the antioxidant potential of this strain. Hence, the strain was further examined for its antioxidant potential using ABTS, metal chelating, and SOD activity assays. According to the results of antioxidant assays, it can be observed that MUSC 93J^T^ extract exhibited significant free radical scavenging activity (Table 3). In ABTS assay, ABTS radical cation was produced by the reaction between a strong oxidizing agent potassium persulfate with ABTS salt and the ability of antioxidant to scavenge the ABTS radical generated in the aqueous phase will be measured (Shalaby and Shanab, 2013). The results showed that MUSC 93J^T^ extract was capable of scavenging 11.80 ± 3.75% of ABTS radicals at the highest test concentration of 2 mg/mL. Besides, MUSC 93J^T^ extract exhibited metal chelating activity of 50.06 ± 1.95% at 2 mg/mL concentration. This indicated the antioxidative potential of MUSC 93J^T^ extract through prevention of transition metals from enhancing the production of ROS (Ser et al., 2016b). In addition, the SOD assay also confirmed the antioxidant potential of MUSC 93J^T^ extract. In SOD assay, the superoxide anion scavenging activity of this extract was determined by the 2-(4-iodophenyl)-3-(4-nitrophenyl)-5-(2,4-disulfophenyl)-2*H*-tetrazolium, monosodium salt (WST) reduction method. The superoxide anion radical produced from hypoxanthine-xanthine oxidase reaction reduces WST-1 to produce yellow formazan (Dudonné et al., 2009; Tan et al., 2015). MUSC 93J^T^ extract exhibited superoxide dismutase (SOD)-like activity which may subsequently prevent the formation of yellow WST-1 formazan. The SOD-like activity of this extract was 83.32 ± 2.62% at the highest tested concentration of 2 mg/mL. All of these assays revealed significant antioxidant potential of MUSC 93J^T^ extract and thus suggested the presence of antioxidant(s) in it.

**Table 3:** **Radical scavenging activity of MUSC 93J^**T**^ evaluated using ABTS, metal chelating, and SOD assays**.

**Antioxidants assays**	**Concentration of MUSC 93J^T^ extract (mg/mL)**	**Mean ± standard error (%)**
ABTS	0.25	5.65 ± 2.56
	0.50	1.35 ± 1.76
	1.00	4.60 ± 3.24
	2.00	11.80 ± 3.75
Metal chelating	0.25	7.86 ± 2.87
	0.50	18.10 ± 2.05
	1.00	33.02 ± 1.07
	2.00	50.06 ± 1.95
SOD	0.25	36.02 ± 3.89
	0.50	51.55 ± 3.54
	1.00	70.29 ± 2.76
	2.00	83.32 ± 2.62

### Cytotoxic activity of strain MUSC 93J^T^ extract

**Figure 3:**
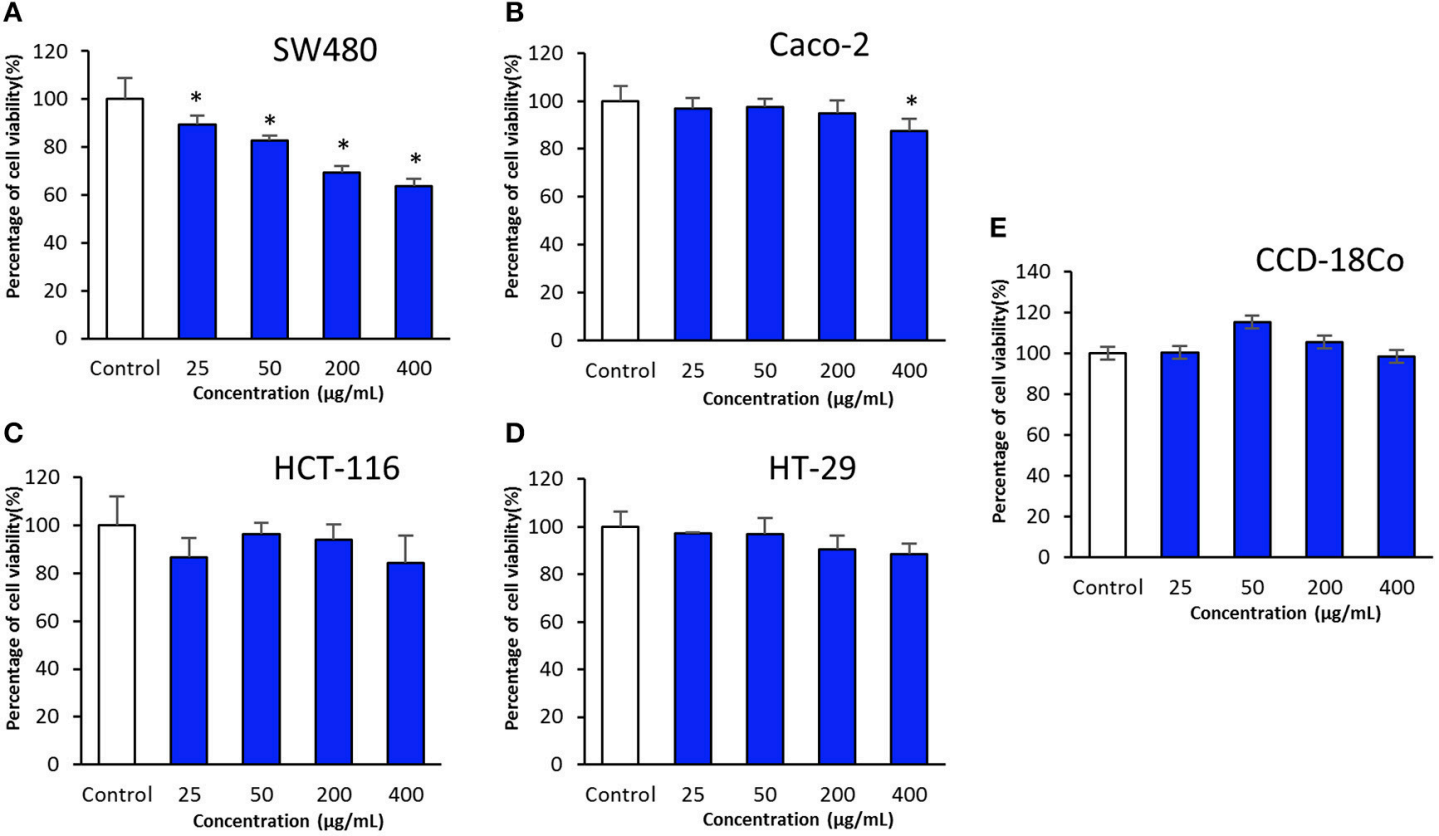
**Cytotoxic activity of MUSC 93J^**T**^ extract against human colon cancer and normal cell lines**. The measurement of cell viability was done using MTT assay. The graphs show cytotoxicity effect of MUSC 93J^T^ extract against **(A)** SW480, **(B)** Caco-2, **(C)** HCT-116, **(D)** HT-29, and **(E)** CCD-18Co. All data are expressed as mean ± standard deviation and significance level are set as 0.05. Symbol (^*^) indicates *p* < 0.05 significant difference between the cells treated with MUSC 93JT extract and control (without MUSC 93JT extract).

In present study, cytotoxic potential of MUSC 93J^T^ extract was examined on human colon cancer cell lines namely HCT-116, HT-29, Caco-2, and SW480 by using the MTT assay. MTT assay is a tetrazolium-based colorimetric assay which operates by measuring the mitochondrial activity in living cells only. The activity of mitochondrial dehydrogenase enzyme of viable cells will transform the MTT tetrazolium salt (yellow) to MTT formazan crystal (purple) (Gerlier and Thomasset, 1986; Ser et al., 2015a; Tan et al., 2015). The use of different type of human colon cancer cell lines with different molecular characteristics as the panels for this experimentation is to assess varying efficacy of cytotoxic activity toward different genetic makeup of cancer cell lines (Tan et al., 2015). The tested results of MUSC 93J^T^ extract against tested colon cancer cell lines were shown in (Figure 3).

The results revealed that MUSC 93J^T^ extract showed varying levels of cytotoxicity against HCT-116, HT-29, Caco-2, and SW480. It was found that the extract exhibited highest cytotoxic effect on SW480 with cell viability recorded at 63.6 ± 3.0% when tested with highest concentration of 400 μg/mL, followed by HCT-116 with cell viability 84.3 ± 11.5% (at concentration 400 μg/mL), then Caco-2 with cell viability 87.5 ± 5.3% (at concentration 400 μg/mL), and lastly the lowest cytotoxic effect on HT-29 with cell viability 88.4 ± 4.4% (at concentration 400 μg/mL; Figure 3). Furthermore, it can be observed that there was a dose dependent effect when the extract was tested against SW480 cells. SW480 cells were significantly inhibited (*p* < 0.05) by increased concentration of the extract. As for the human normal colon CCD-18Co cells, no significant cytotoxic effect was exerted by MUSC 93J^T^ extract against these cells (Figure 3). Overall, the results suggested that MUSC 93J^T^ extract is more cytotoxic toward the colon cancer cells lines than the normal colon cells with particularly stronger cytotoxic activity against colon cancer cell line SW480.

### GC-MS analysis of strain MUSC 93J^T^ extract

**Figure 4:**
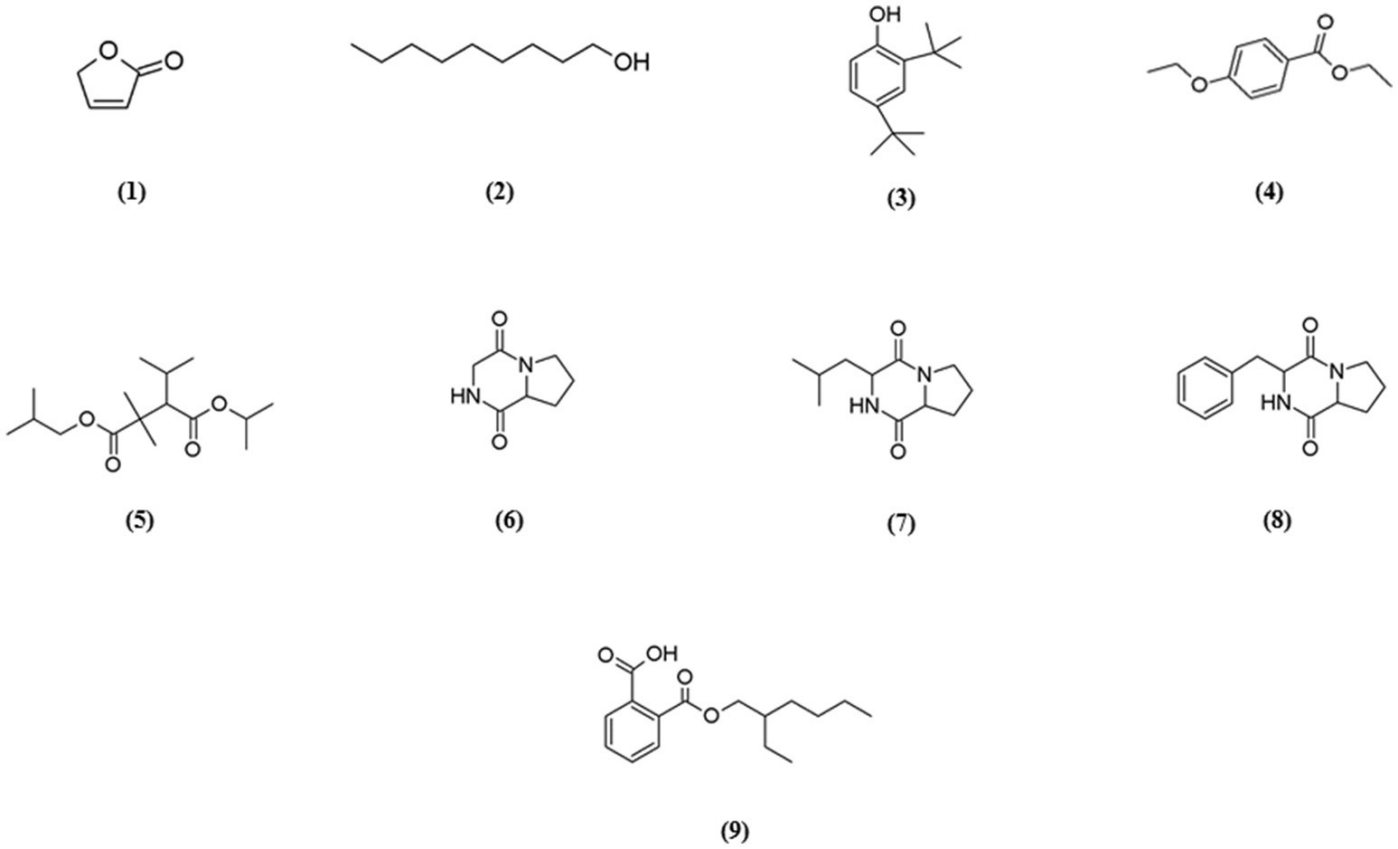
**Chemical structures of constituents detected in MUSC 93J^**T**^ extract**.

GC-MS analysis was conducted to aid in chemical profiling and to identify compounds that present in the extract. The results of GC-MS analysis revealed that strain MUSC 93J^T^ extract contains nine compounds (Table 4): 2(5H)-Furanone **(1)**, 1-Nonanol **(2)**, Phenol, 2,4-bis (1,1-dimethylethyl)-**(3)**, Benzoic acid, 4-ethoxy-, ethyl ester **(4)**, Pentanoic acid, 2,2,4-trimethyl-3-carboxyisopropyl, isobutyl ester **(5)**, Pyrrolo[1,2-a]pyrazine-1,4-dione, hexahydro- **(6)**, Pyrrolo[1,2-a]pyrazine-1,4-dione, hexahydro-3-(2-methylpropyl)- **(7)**, Pyrrolo[1,2-a]pyrazine-1,4-dione, hexahydro-3-(phenylmethyl)- **(8)**, 1,2-Benzenedicarboxylic acid, mono(2-ethylhexyl) ester **(9)** with chemical structures as shown in Figure 4. From this analysis, butenolides, fatty alcohol, phenolic, benzoic acid esters, hydrocarbon, pyrrolopyrazine, and dicarboxylic acid ester were the main classes of compounds present in strain MUSC 93J^T^ extract.

**Table 4:** **Compounds identified from MUSC 93J^**T**^ extract using GC-MS**.

**No**.	**Retention time (min)**	**Compound**	**Class**	**Molecular formula**	**Molecular weight (MW)**	**Quality (%)**
1	13.787	2(5H)-Furanone	Butenolides	C_4_H_4_O_2_	84	83
2	27.325	1-Nonanol	Fatty alcohol	C_9_H_20_O	144	83
3	44.457	Phenol, 2,4-bis(1,1-dimethylethyl)-	Phenolic compound	C_14_H_22_O	206	93
4	44.892	Benzoic acid, 4-ethoxy-, ethyl ester	Benzoic acid esters	C_11_H_14_O_3_	194	91
5	47.758	Pentanoic acid, 2,2,4-trimethyl-3-carboxyisopropyl, isobutyl ester	Hydrocarbon	C_16_H_30_O_4_	286	78
6	53.165	Pyrrolo[1,2-a]pyrazine-1,4-dione, hexahydro-	Pyrrolopyrazine	C_7_H_10_N_2_O_2_	154	96
7	59.076	Pyrrolo[1,2-a]pyrazine-1,4-dione, hexahydro-3-(2-methylpropyl)-	Pyrrolopyrazine	C_11_H_18_N_2_O_2_	210	83
8	72.031	Pyrrolo[1,2-a]pyrazine-1,4-dione, hexahydro-3-(phenylmethyl)-	Pyrrolopyrazine	C_14_H_16_N_2_O_2_	244	80
9	76.883	1,2-Benzenedicarboxylic acid, mono(2-ethylhexyl) ester	Dicarboxylic acid ester	C_16_H_22_O_4_	278	87

Pyrrolopyrazines are capable of exerting a variety of bioactivities such as antioxidant, antitumor, anti-angiogenesis, and antimicrobial (Ser et al., 2015a,b). According to the GC-MS analysis, three pyrrolopyrazine compounds were detected in the MUSC 93J^T^ extract, including Pyrrolo[1,2-a]pyrazine-1,4-dione, hexahydro- **(6)**, Pyrrolo[1,2-a]pyrazine-1,4-dione, hexahydro-3-(2-methylpropyl)- **(7)**, and Pyrrolo[1,2-a]pyrazine-1,4-dione, hexahydro-3-(phenylmethyl)- **(8)**. In previous studies, pyrrolo[1,2-a]pyrazine-1,4-dione, hexahydro- **(6)** was reported to be present in *Streptomyces mangrovisoli*, a novel *Streptomyces* species isolated from mangrove forest in Malaysia by Ser et al. (2015b) and it was suggested that this compound may be responsible for the antioxidant activity of this species. In addition, Pyrrolo[1,2-a]pyrazine-1,4-dione, hexahydro- **(6)** was also found in *Streptomyces pluripotens* and *Bacillus* sp. with the capability to reduce oxidative damages by free radicals (Gopi et al., 2014; Ser et al., 2015a). Moreover, Pyrrolo[1,2-a]pyrazine-1,4-dione, hexahydro-3-(2-methylpropyl)- **(7)** was detected in *Streptomyces* sp. VITMK1 isolated from India mangrove soil by Manimaran et al. (2015). Mithun and Rao (2012) also reported the detection of Pyrrolo[1,2-a]pyrazine-1,4-dione, hexahydro-3-(2-methylpropyl)- **(7)** in *Micrococcus luteus* with promising anticancer activity on HCT15. Also, all three pyrrolopyrazine compounds: Pyrrolo[1,2-a]pyrazine-1,4-dione, hexahydro- **(6)**, Pyrrolo[1,2-a]pyrazine-1,4-dione, hexahydro-3-(2-methylpropyl)- **(7)**, and Pyrrolo[1,2-a]pyrazine-1,4-dione, hexahydro-3-(phenylmethyl)- **(8)** were detected in *Streptomyces* sp. MUM 256 isolated from mangrove forest in Malaysia by Tan et al. (2015) which exhibited antioxidant and anticancer activities that could be due to the presence of these pyrrolopyrazines. Hence, the antioxidant and cytotoxic activities exhibited by MUSC 93J^T^ extract could be mainly due to the presence of these pyrrolopyrazine compounds.

Besides, phenolic compounds have been regard as important antioxidant agents responsible in scavenging ROS (Narendhran et al., 2014). Phenol, 2,4-bis(1,1-dimethylethyl)- **(3)** was the phenolic compound detected in strain MUSC 93J^T^ extract. Some of the recent studies have demonstrated the presence of phenol, 2,4-bis(1,1-dimethylethyl)- in *Streptomyces* bacteria through GC-MS analysis. For instance, Narendhran et al. (2014) successfully detected phenol, 2,4-bis(1,1-dimethylethyl)- in *Streptomyces cavouresis* KUV39 isolated from vermicompost samples collected in India. The study also presented that this compound could be responsible for the antioxidant and cytotoxic properties of *Streptomyces cavouresis* KUV39. Besides, phenol, 2,4-bis(1,1-dimethylethyl)- was also detected in *Streptomyces* sp. MUM256 with potential antioxidant activity in the study conducted by Tan et al. (2015). A recently discovered novel *Streptomyces antioxidans* by Ser et al. (2016c) reported the detection of phenol, 2,4-bis(1,1-dimethylethyl)- was detected in the extract, which may had contributed to the strain's free radical scavenging activities.

The compound 1,2-Benzenedicarboxylic acid, mono(2-ethylhexyl) ester **(9)** detected in MUSC 93J^T^ extract has been previously reported to possess potential antibacterial, antifungal, and cytotoxic activities (Saxena et al., 2015; Tan et al., 2015). This compound was also detected in other *Streptomyces* sp., with cytotoxic activity against several cancer cell lines such as liver cancer cell line HepG2 and breast cancer cell line MCF7 (Krishnan et al., 2014).

Lastly, other detected compounds in MUSC 93J^T^ including 2(5H)-Furanone **(1)**, 1-Nonanol **(2)**, Benzoic acid, 4-ethoxy-, ethyl ester **(4)**, and Pentanoic acid, 2,2,4-trimethyl-3-carboxyisopropyl, isobutyl ester **(5)** were also previously found to be present in microbes. For instance, 2(5H)-Furanone was detected in *Lactobacillus helveticus* (Ndagijimana et al., 2006), 1-Nonanol was detected in *Streptomyces albidoflavus* (Sunesson et al., 1997), Benzoic acid, 4-ethoxy-, ethyl ester was detected in *Bacillus* sp. (Mishra and Thakur, 2016), and Pentanoic acid, 2,2,4-trimethyl-3-carboxyisopropyl, isobutyl ester was detected in *Aspergillus carbonarius* ITEM 5010 (Sinha et al., 2015).

Overall, most of the chemical compounds identified by GC-MS are well-known for their antioxidant and anticancer activities. Therefore, we propose that these compounds, either alone or in combination, might be the main contributing factors for the antioxidant and cytotoxic properties present in MUSC 93J^T^ extract.

### Description of *Streptomyces colonosanans* sp. nov.

*Streptomyces colonosanans* (co.lo.no.sa'nans. Gr. n. kolon, intestine, colon; L. part. adj. sanans, healing; N.L. part. adj. colonosanans, colon-healing).

Cells stain Gram-positive, forming light yellow aerial and vivid yellow substrate mycelium on ISP 2 agar. The colors of the aerial and substrate mycelium are media-dependent (Table S1). Cells grow well on ISP 2 agar, ISP 6 agar, and *Streptomyces* agar after 7 days at 28°C, cells grow moderately on starch casein agar, and cells grow weakly on nutrient agar and did not grow on actinomycetes isolation agar, ISP 3, ISP 4, ISP 5, and ISP 7 agar. Cells grow at 0–4% NaCl tolerance (optimum 0–2%), 24–36°C (optimum 28–32°C), at pH 6.0–7.0 (optimum pH 6.0). Cells are positive for catalase and hemolytic activities. Hydrolysis of soluble starch, casein and tributyrin (lipase) are positive; but negative for hydrolysis of chitin carboxymethylcellulose and xylan.

The following compounds are utilized as sole carbon sources: α-D-glucose, α-hydroxy-butyric acid, β-hydroxyl-D,L-butyric acid, D-cellobiose, dextrin, D-fructose-6-phosphate, L-fucose, D-galactose, D-galacturonic acid, D-glucose-6-phosphate, D-gluconic acid, D-glucuronic acid, D-mannose, D-melibiose, D-raffinose, D-trehalose, gelatin, gentiobiose, glucuronamide, glycerol, L-galactonic acid lactone, L-lactic acid, p-hydroxy-phenylacetic acid, Tween 40, γ-amino-butyric acid, acetoacetic acid, and α-keto-butyric acid. The following compounds are not utilized as sole carbon sources: acetic acid, α-D-lactose, α-keto-glutaric acid, β-methyl-D-glucoside, bromo-succinic acid, citric acid, D-arabitol, D-aspartic acid, D-fructose, D-fucose, D-lactic acid methyl ester, D-malic acid, D-maltose, D-mannitol, D-saccharic acid, D-salicin, D-serine, D-sorbitol, D-turanose, formic acid, glycyl-L-proline, inosine, L-malic acid, L-rhamnose, methyl pyruvate, mucic acid, N-acetyl-β-D-mannosamine, N-acetyl-D-galactosamine, N-acetyl-D-glucosamine, N-acetyl-neuraminic acid, pectin, propionic acid, quinic acid, stachyose, sucrose, myo-inositol, and 3-methyl glucose. The following compounds are not utilized as sole nitrogen sources: L-alanine, L-arginine, L-aspartic acid, L-glutamic acid, L-histidine, L-pyroglutamic acid, and L-serine.

The cell wall peptidoglycan contains LL-diaminopimelic acid. The predominant menaquinones were identified as MK-9(H_8_) and MK-9(H_6_). The whole cell sugars are glucose, mannose and ribose. The polar lipids consist of aminolipid, diphosphatidylglycerol, lipid, phospholipid, phosphatidylinositol, phosphatidylethanolamine, and phosphoglycolipid. The predominant cellular fatty acids (>10.0%) are anteiso-C_15:0_, C_16:0_, and iso-C_16:0_.

The type strain is MUSC 93J^T^ (= DSM 102042^T^ = MCCC 1K02298^T^), isolated from mangrove soil collected from mangrove forest located in Kuching, state of Sarawak, Malaysia. The 16S rRNA gene sequence of strain MUSC 93J^T^ has been deposited in GenBank/EMBL/DDBJ under the accession number KP998434. The genome of MUSC 93J^T^ consists of 7,015,076 bp with average coverage of 53.0-fold and the G + C content of the genomic DNA of the type strain is 69.90 mol%. The whole project of MUSC 93J^T^ has been deposited at DDBJ/EMBL/GenBank under accession number MLYP00000000. A total of 5,859 coding genes was predicted and assigned to 402 subsystems, along with 66 tRNA and 5 RNA genes (Table 5). Based on RAST annotation, most of the genes were involved in amino acids and derivatives metabolism (9.18%), followed by carbohydrates metabolism (6.21%) and protein metabolism subsystems (4.91%).

**Table 5:** **General features of ***Streptomyces colonosanans*** MUSC 93J^**T**^ genome**.

	**Streptomyces colonosanans MUSC 93J^T^**
Genome size (bp)	7,015,076
Contigs	166
Contigs N_50_ (bp)	99,963
G + C content %	69.90
Protein coding genes	5,859
tRNA	66
rRNA	3 (5S),1 (16S),1 (23S)

## Conclusion

This study has revealed that strain MUSC 93J^T^ is a novel species of the genus *Streptomyces*, isolated from the soil of mangrove forest located in Kuching, Sarawak. The name *Streptomyces colonosanans* sp. nov. is proposed and the type strain is MUSC 93J^T^ (= DSM 102042^T^ = MCCC 1K02298^T^). This study revealed that the extract of strain MUSC 93J^T^ has significant antioxidant potential with radical scavenging activity up to 83.32 ± 2.62% via SOD assay. Additionally, this strain exhibited cytotoxic activity against several human colon cancer cell lines, with highest cytotoxic effect on SW480 with cell viability of 63.6 ± 3.0%. Chemical analysis via GC-MS further confirms that the strain is capable of producing chemo-preventive related metabolites. Hence, this study demonstrated the biopharmaceutical potential of novel strain *Streptomyces colonosanans* MUSC 93J^T^ with capability to produce bioactive compounds responsible for the antioxidant and cytotoxic activities. Strain MUSC 93J^T^ serves as a potentially high quality resource for drug discovery and further studies pertaining the development of chemo-preventive drugs from this strain are highly valuable.

## Author contributions

The experiments, data analysis, and manuscript writing were performed by JL and HS, while AD, SS, SIB, TK, NM, K-GC, BG, and LL provided vital guidance, insight and technical support for the completion of the project. L-HL and BG founded the research project.

### Conflict of interest statement

The authors declare that the research was conducted in the absence of any commercial or financial relationships that could be construed as a potential conflict of interest.
