# Supplementary material for: Streptomyces colonosanans sp. nov., A Novel Actinobacterium Isolated from Malaysia Mangrove Soil Exhibiting Antioxidative Activity and Cytotoxic Potential against Human Colon Cancer Cell Lines
Source: Front Microbiol. 2017 May 16;8:877. doi: 10.3389/fmicb.2017.00877 (PMC5432915; doi:10.3389/fmicb.2017.00877)
Supplement: Supplementary file 1 [file Table1.pdf]

Supplementary Material

Jodi Woan-Fei Law[1], Hooi-Leng Ser[1], Acharaporn Duangjai[2,3], Surasak Saokaew[1,3,4], Sarah Ibrahim Bukhari[5], Tahir Mehmood Khan[1,6], Nurul-Syakima Ab Mutalib[7], Kok-Gan Chan[8], Bey-Hing Goh[1,3*], Learn-Han Lee[1,3*]

[1]Novel Bacteria and Drug Discovery Research Group, School of Pharmacy, Monash University Malaysia, Bandar Sunway, Malaysia
[2]Division of Physiology, School of Medical Sciences, University of Phayao, Phayao, Thailand
[3]Center of Health Outcomes Research and Therapeutic Safety (Cohorts), School of Pharmaceutical Sciences, University of Phayao, Phayao, Thailand
[4]Faculty of Pharmaceutical Sciences, pharmaceutical Outcomes Research Center (CPOR), Naresuan University, Phitsanulok, Thailand
[5]Department of Pharmaceutics, College of Pharmacy, King Saud University, Riyadh, Saudi Arabia
[6]Department of Pharmacy, Absyn University Peshawar, Peshawar, Pakistan,
[7]UKM Medical Molecular Biology Institute (UMBI), UKM Medical Centre, University Kebangsaan Malaysia, Kuala Lumpur, Malaysia
[8]Division of Genetics and Molecular Biology, Institute of Biological Sciences, Faculty of Science, University of Malaya, Kuala Lumpur, Malaysia

*** Correspondence:** Learn-Han Lee, Novel Bacteria and Drug Discovery Research Group, School of Pharmacy, Monash University Malaysia, 47500 Bandar Sunway, Selangor Darul Ehsan, Malaysia. lee.learn.han@monash.edu; leelearnhan@yahoo.com; Bey-Hing Goh, Novel Bacteria and Drug Discovery Research Group, School of Pharmacy, Monash University Malaysia, 47500 Bandar Sunway, Selangor Darul Ehsan, Malaysia. goh.bey.hing@monash.edu.

Figure S1. Maximum-likelihood tree based on almost complete 16S rRNA sequences (1490 nucleotides) showing relationship between strain MUSC 93J^{T} and representatives of some other related taxa. Bootstrap values (>50%) based on 1000 re-sampled datasets are shown at branch nodes. Bar, 0.001 substitutions per site. Asterisks indicate that the corresponding nodes were also recovered using neighbour-joining tree-making algorithms.

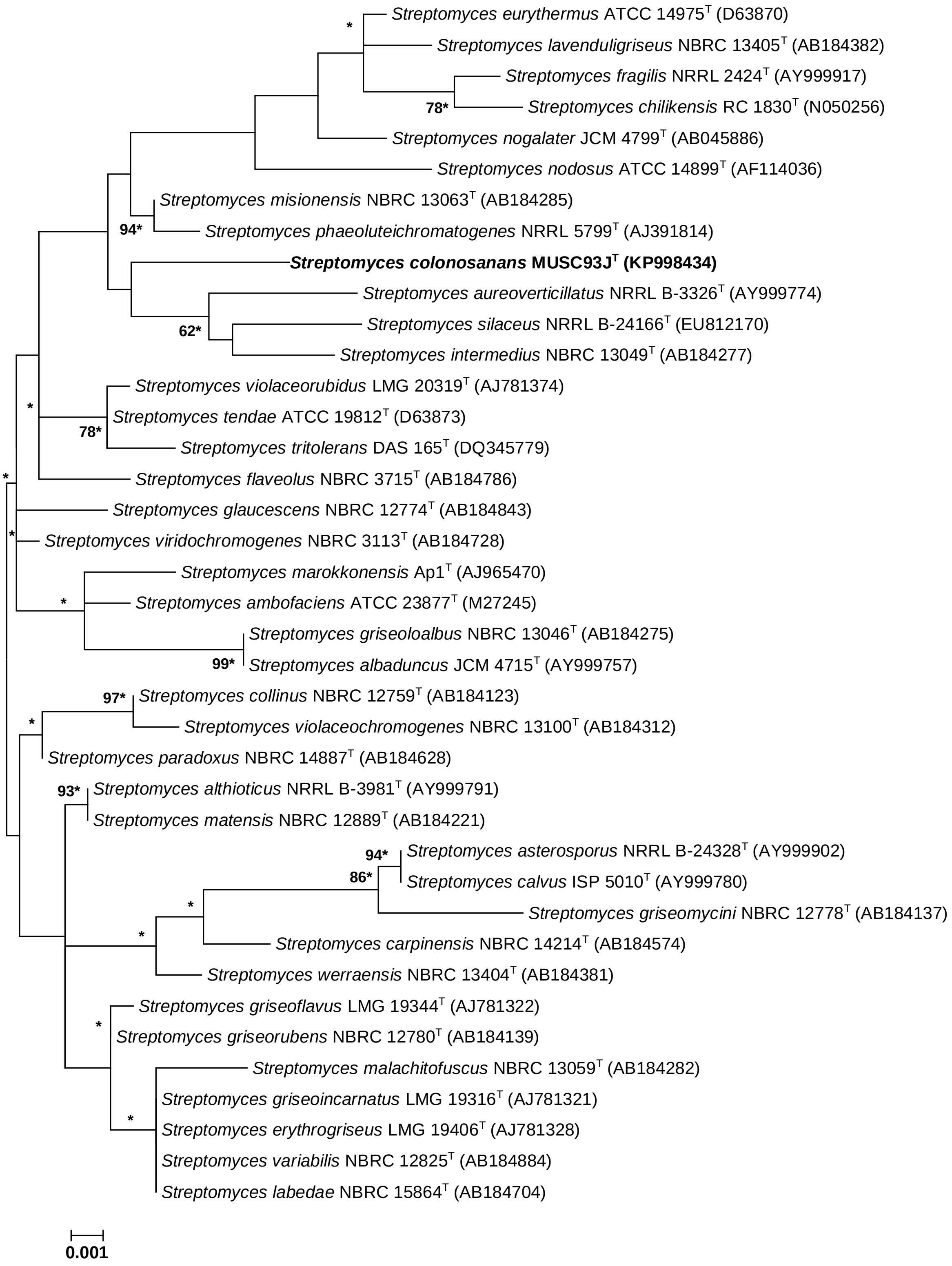

Figure S2. BOX-PCR comparison of strain MUSC 93J^{T} and the closest related type strains. Strains: 1, *Streptomyces colonosanans* MUSC 93J^{T}; 2, *Streptomyces malachitofuscus* JCM 4493^{T}; 3, *Streptomyces misionensis* NBRC 13063^{T}; 4, *Streptomyces phaeoluteichromatogenes* DSM 41898^{T}; M, GeneRuler 1kb DNA ladder marker.

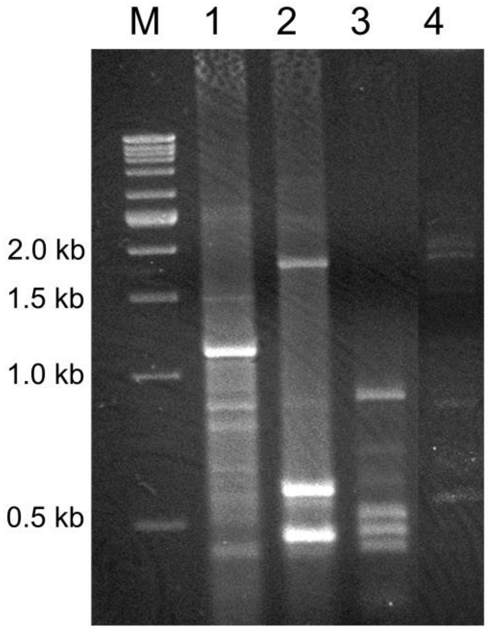

Figure S3. Two dimensional total lipid profile of strain MUSC 93J^T and *Streptomyces malachitofuscus* JCM 4493^T.

AL, aminolipid; DPG, diphosphatidylglycerol; PE, phosphatidylethanolamine; PGL, phosphoglycolipid; PI, phosphatidylinositol; PL, phospholipid; L, lipid.

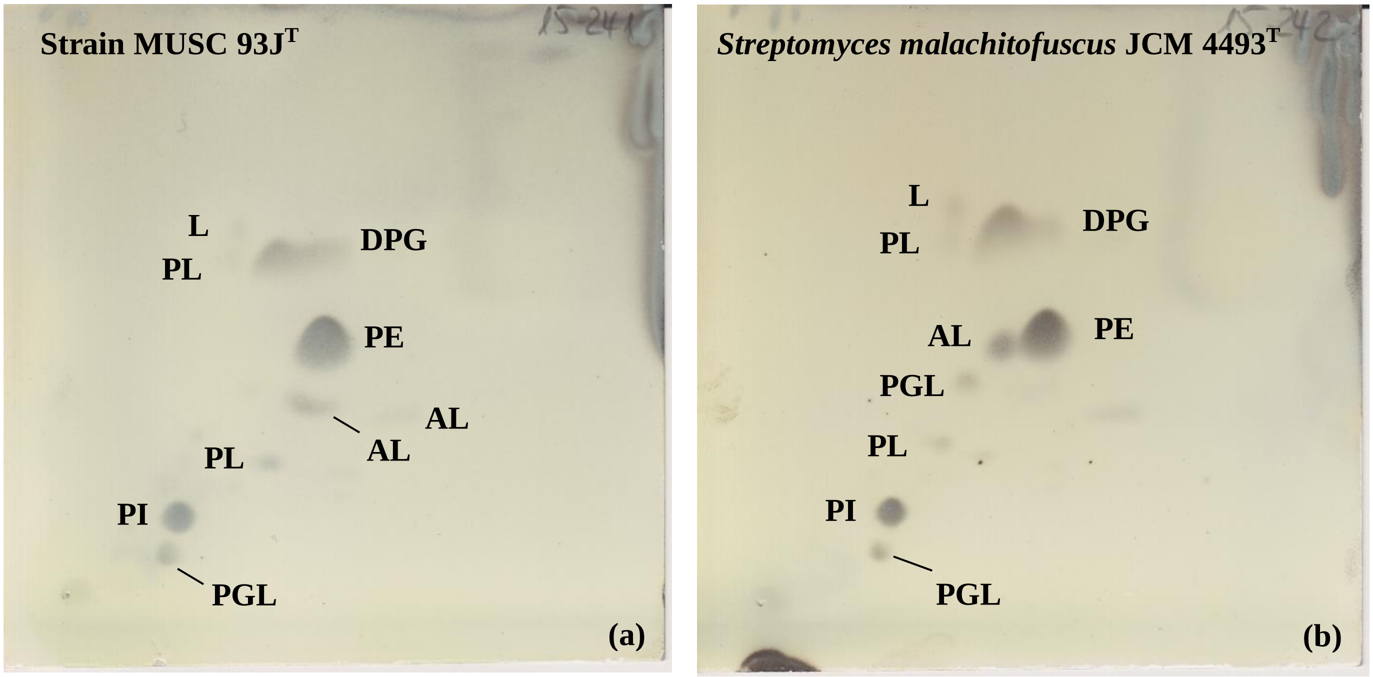

Table S1. Cultural characteristics of strain MUSC 93J^{T} on different media at 28 ^{0}C after 7-14 days of incubation.

-, Not detected

Medium	Growth	Colony color	
		**Aerial mycelium**	**Substrate mycelium**
Yeast malt agar (ISP 2)	Good	Light yellow	Vivid yellow
Oat Meal agar (ISP 3)	No growth	-	-
Inorganic Salt Starch agar (ISP 4)	No growth	-	-
Glycerol Asparagine Agar Base (ISP 5)	No growth	-	-
Peptone Yeast Extract Iron agar (ISP 6)	Good	Moderate yellow	Brilliant yellow
Tyrosine agar base (ISP 7)	No growth	-	-
Streptomyces agar	Good	Brilliant greenish yellow	Vivid greenish yellow
Starch casein agar	Moderate	Greenish yellow	Yellowish white
Actinomycete isolation agar	No growth	-	-
Nutrient agar	Weak	Pale greenish yellow	Yellowish white
